# Supplementary figures and images for: Treatment with galectin-1 improves myogenic potential and membrane repair in dysferlin-deficient models
Source: PLoS One. 2020 Sep 3;15(9):e0238441. doi: 10.1371/journal.pone.0238441 (PMC7470338; doi:10.1371/journal.pone.0238441)

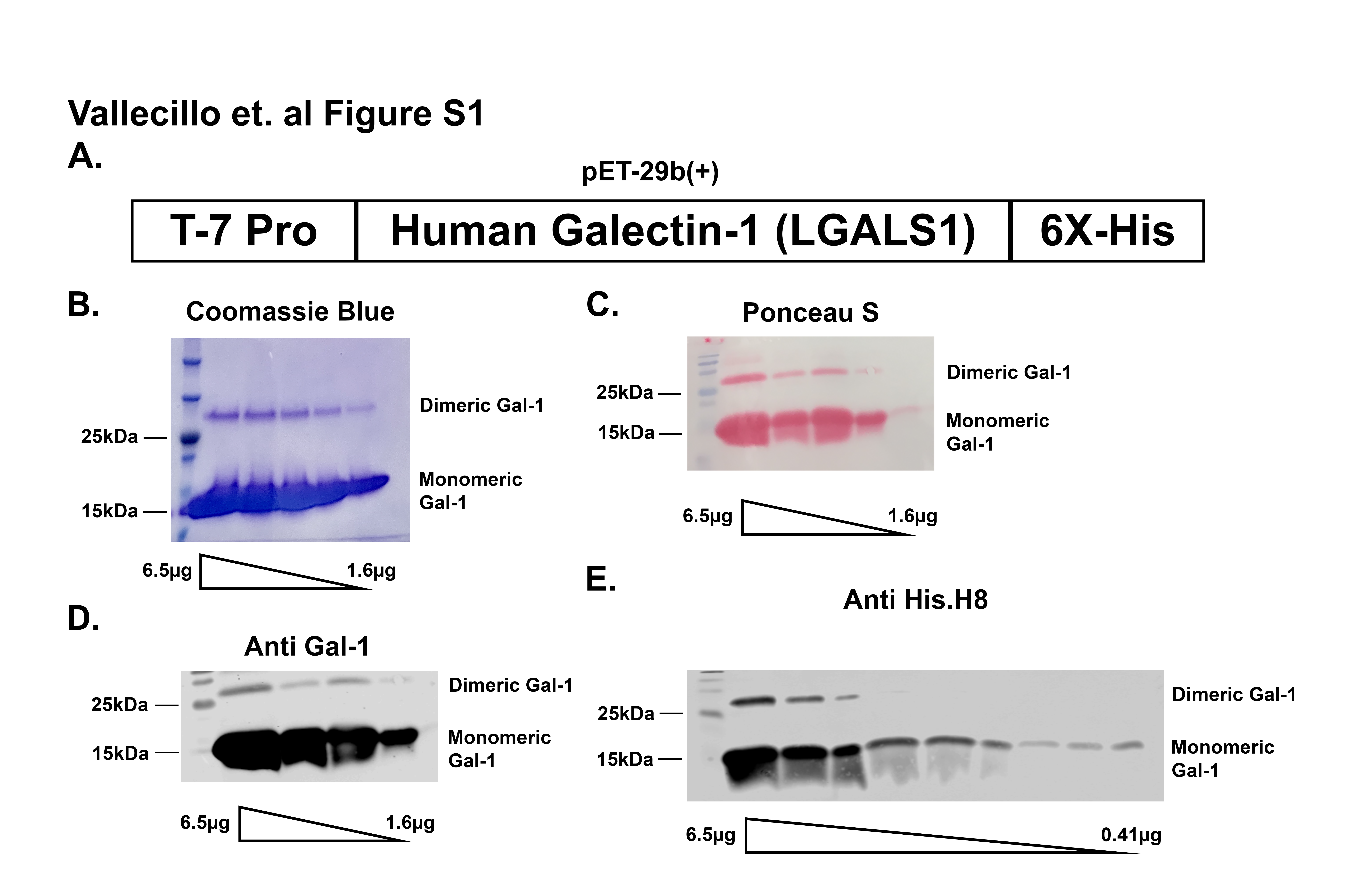

Supplement: S1 Fig — A. rHsGal-1 construct which was inserted into pET-29b (+) vector. B. Coomassie Blue Stain of Gal-1. C. Ponceau S stain of Gal-1. D. Western blot image of Anti-Gal-1 at decreasing dosages. E. Western blot image of Anti-6x-His at decreasing dosages. (TIF) [file pone.0238441.s001.tif]

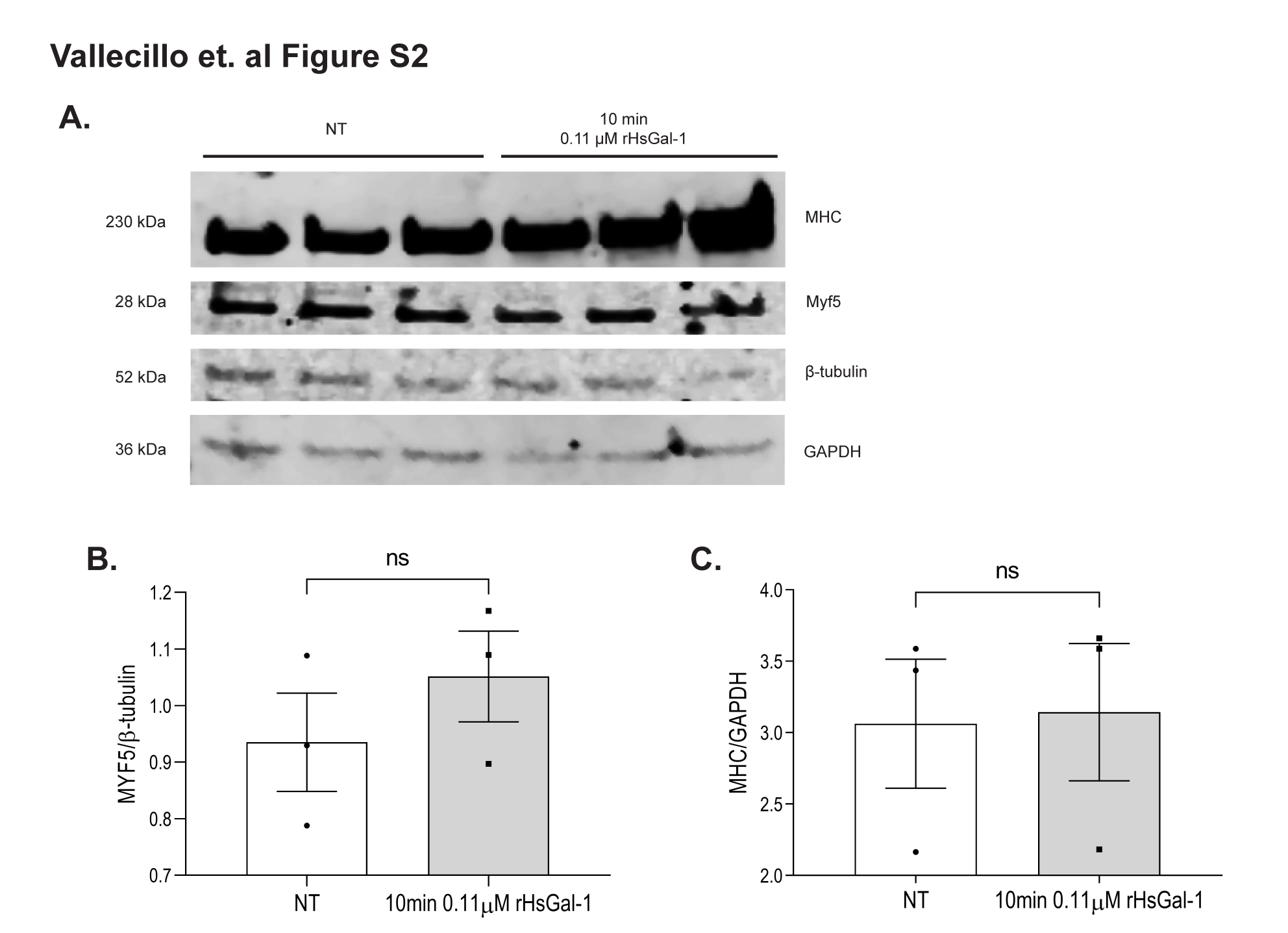

Supplement: S2 Fig — A. Western blot images of NT and 10min 0.11uM rHsGal-1 treated cells. B. Quantification of MYF5 expression. C. Quantification of MHC expression. (TIF) [file pone.0238441.s002.tif]

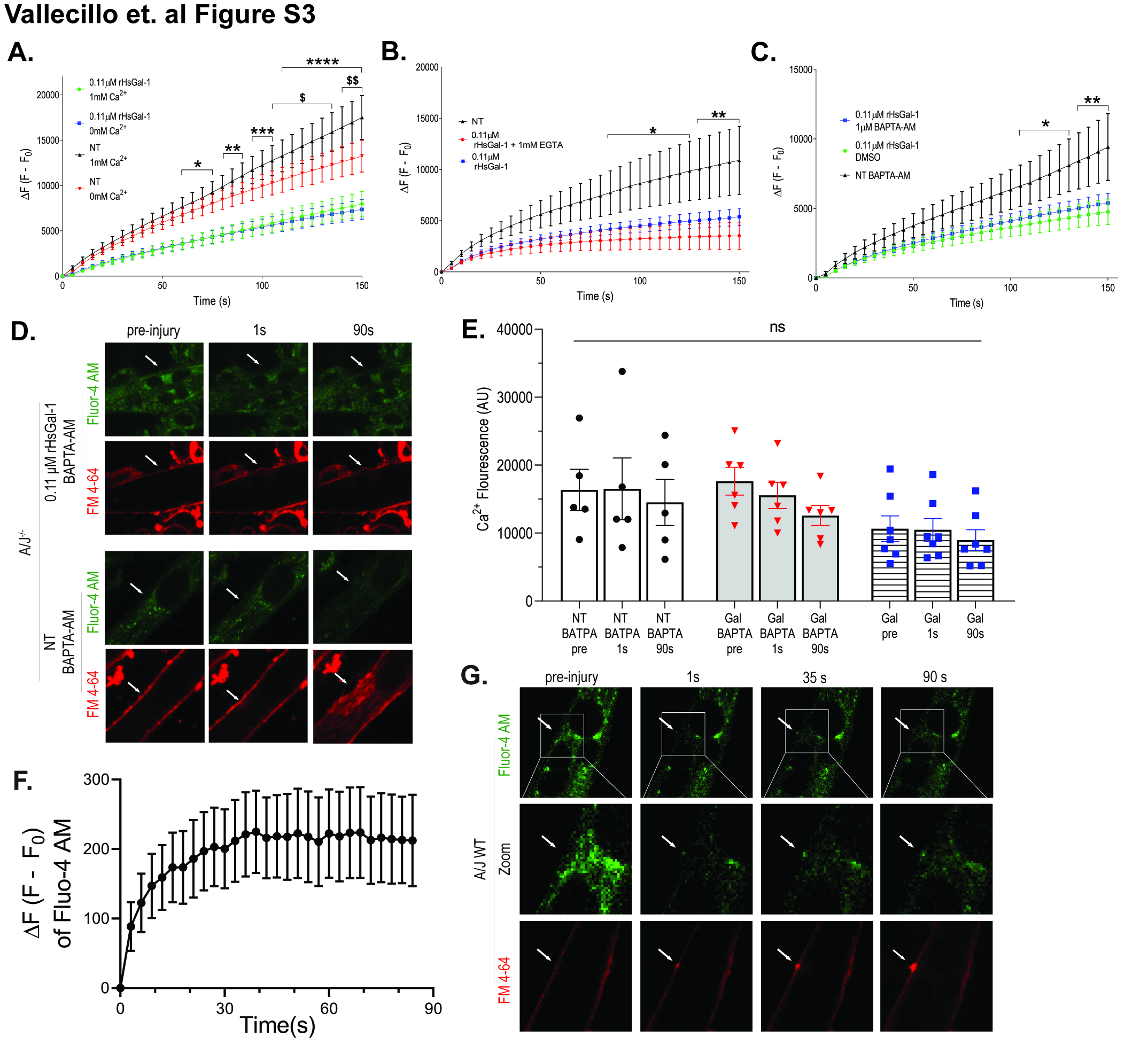

Supplement: S3 Fig — A. Quantification of the change in fluorescent intensity in 0.11 μM rHsGal-1 treated A/J-/- myotube with or without extracellular Ca2+ compared to NT myotubes supplemented or not with extracellular Ca2+. B. Quantification of the change in fluorescent intensity in 0.11 μM rHsGal-1 treated A/J-/- myotube with or without EGTA compared to NT. C. Quantification of the change in fluorescent intensity in 0.11 μM rHsGal-1 treated A/J-/- myotubes with or without BAPTA-AM compared to NT. D. Representative images of NT and rHsGal-1 treated myotubes, with FM4-464 and Fluo-4AM, pre-injury, 1 s after injury, and 90s after injury. White arrows indicate site of injury. E. Quantification of Fluo-4 fluorescence within myotubes pre-injury, 1 s after injury, and 90s after injury. F. Representative images of A/J WT myotubes with FM4-64 and Fluo-4AM pre-injury and 1s, 35s, and 90s after injury. White arrows indicate site of injury. G. Quantification of change in Fluo-4AM fluorescence at injury. p values were measured by 2-way ANOVA multiple comparison test and indicated by *p< 0.05, **p< 0.01, ***p< 0.001, and ****p< 0.0001. Additionally, $p < .05, and $ $p < .01 between NT and 0.11 μM rHsGal-1 0mM Ca2+ treatment. Error bars represent SEM. n > 5 for each group. (TIF) [file pone.0238441.s003.tif]

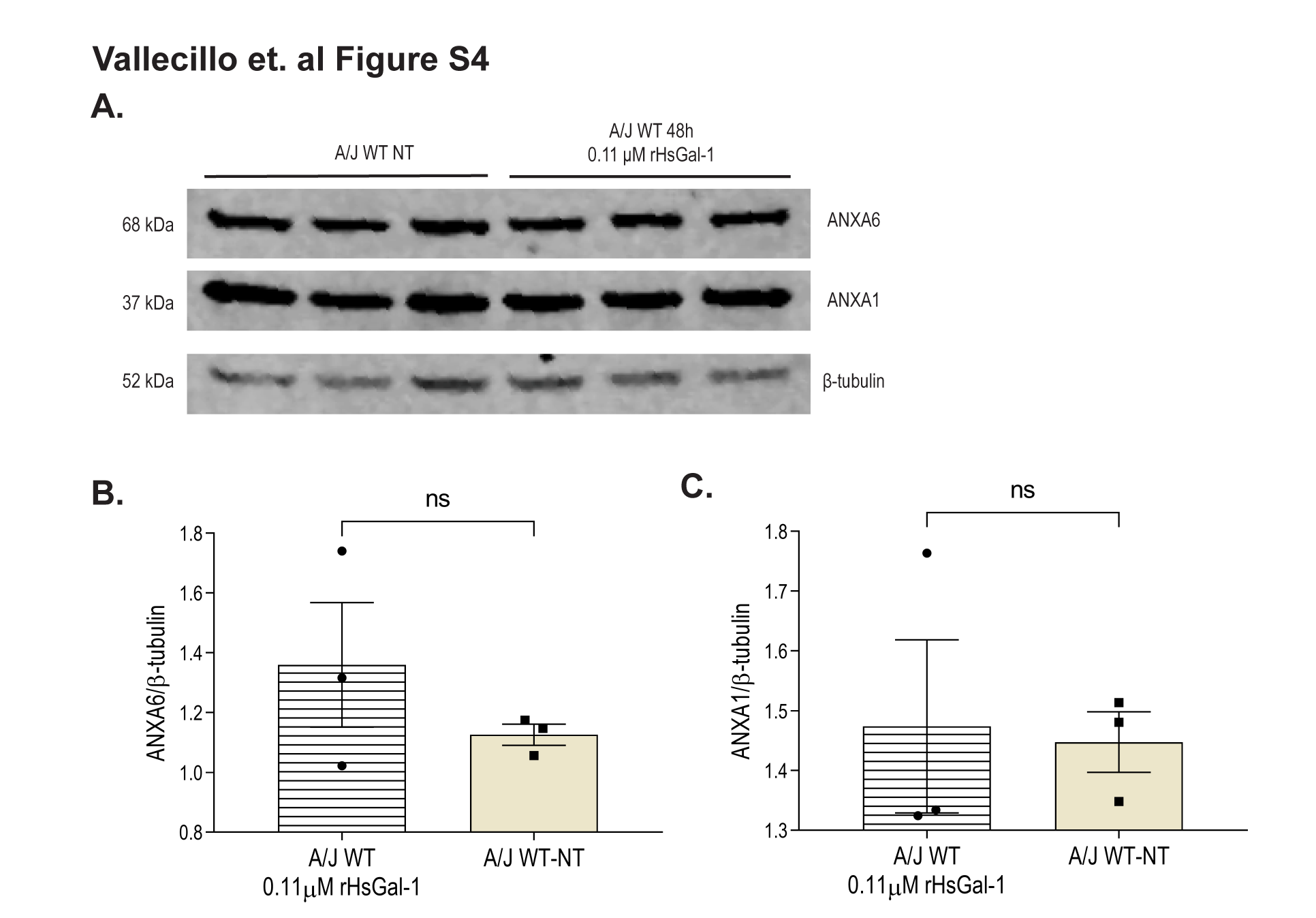

Supplement: S4 Fig — A. Western blot images of A/J+/+ cells after no treatment (NT) or 48hr treatment with 0.11uM rHsGal-1. B. Quantification of ANXA6 expression. C. Quantification of ANAX1 expression. (TIF) [file pone.0238441.s004.tif]

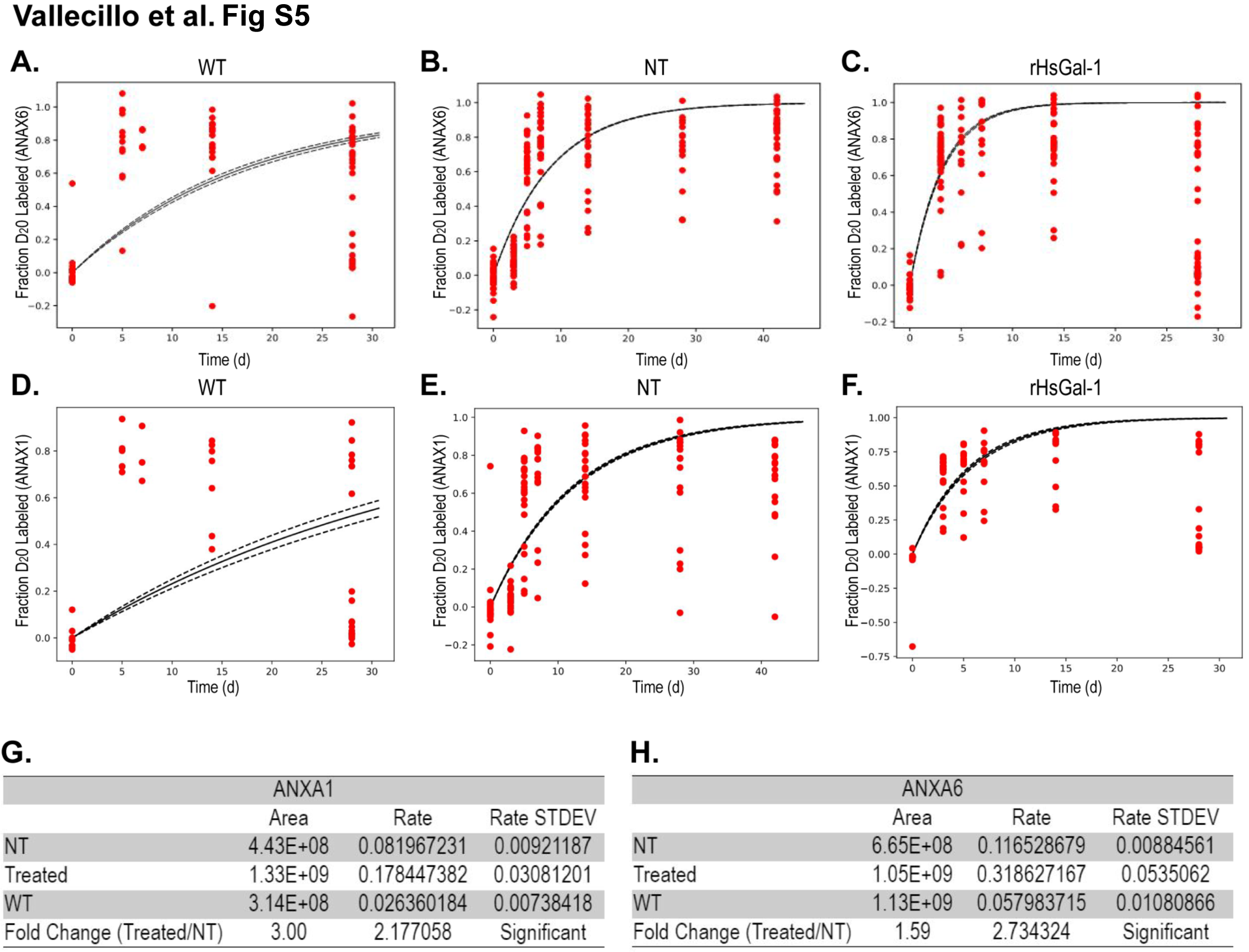

Supplement: S5 Fig — A–C. ANXA6 kinetic graphs quantifying the fraction of ANXA6 peptides incorporating D2O over time in days. D–F. ANXA1 kinetic graphs quantifying the fraction of ANXA1 peptides incorporating D2O over time in days. G. table with ANXA1 area (relative abundance) and turnover rate and its standard deviation. H. table with ANXA6 area (relative abundance) and turnover rate and its standard deviation. (TIF) [file pone.0238441.s005.tif]

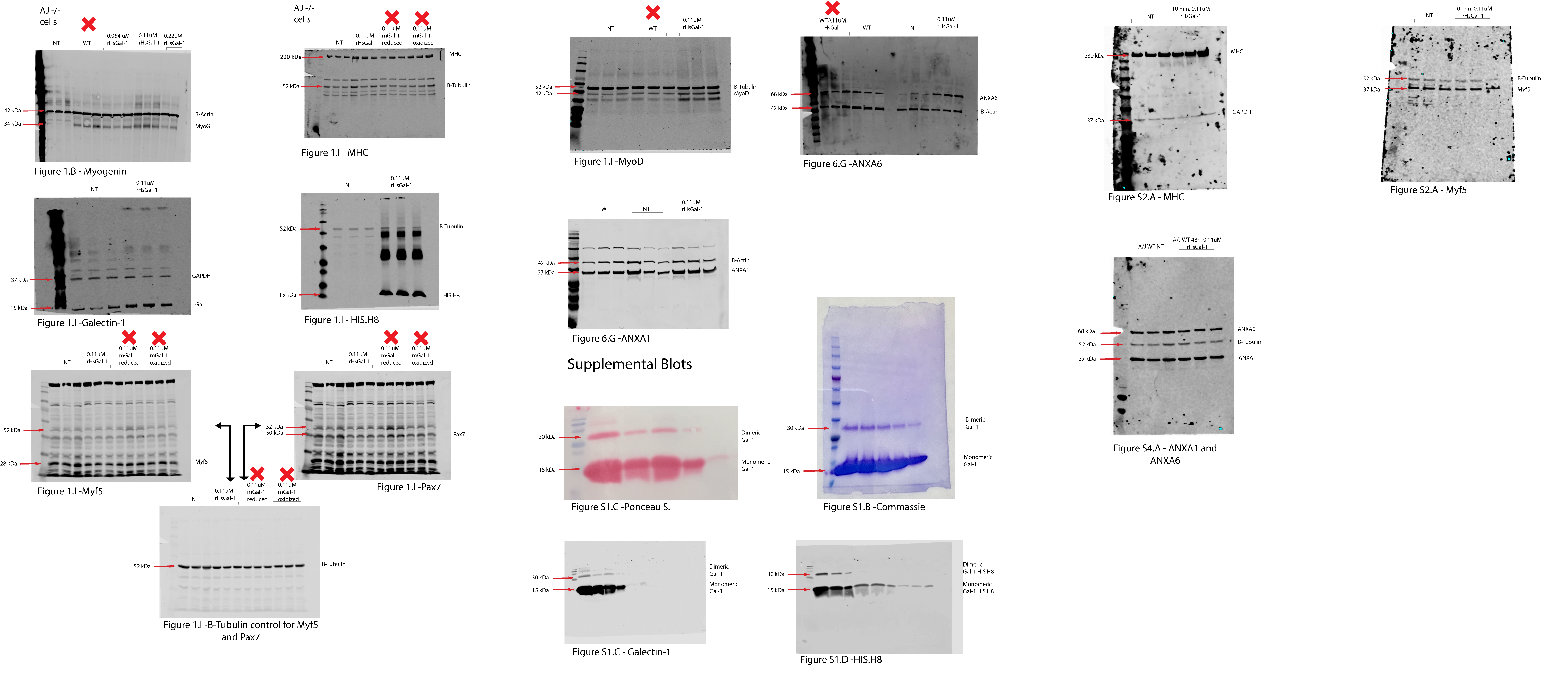

Supplement: S1 Raw images — (TIF) [file pone.0238441.s009.tif]
