# Supplementary material for: Treatment with galectin-1 improves myogenic potential and membrane repair in dysferlin-deficient models
Source: PLoS One. 2020 Sep 3;15(9):e0238441. doi: 10.1371/journal.pone.0238441 (PMC7470338; doi:10.1371/journal.pone.0238441)
Supplement: Attachment — Submitted filename: Response to reviewers PLOS ONE 081220.pdf [file pone.0238441.s011.pdf]

DEPARTMENT OF CHEMISTRY AND BIOCHEMISTRY
BRIGHAM YOUNG UNIVERSITY
C100 BENSON SCIENCE BUILDING
PROVO, UTAH 84602-5700
(801) 422-3667

August 13, 2020

Dear Editors

We thank the reviewer for their generous comments on the manuscript and have edited the manuscript to address their concerns. Our answer to the editors and reviewer concern is in red below the original comment.

We believe that the manuscript in now suitable for publication PLOS ONE.

Feel free to contact me with any questions.

Sincerely,

Dr. Pam M. Van Ry
Assistant Professor
Brigham Young University
Department of Chemistry and Biochemistry
801-422-1540
pvanry@chem.byu.edu
C209, BNSN
Provo, Utah 84602

Reviewer #2:

1. One minor comment is that rearrange Fig 6. If Fig 6 is moved after Fig 3 or 4, story would be stronger since it is showing that enhanced membrane repair of Gal-1 treated Dysf-/-myofibers, which is more physiologically relevant than cultured myotubes.

As suggested, we rearranged the manuscript so that Fig 6 is now Fig 4. All reference to figures in the manuscript have been updated so they coincide with the new figure numbering. Additionally, we have rearranged all relevant paragraphs to strengthen the story.
